# Treatment of Syphilis in Pregnancy and Congenital Syphilis: Current Evidence, Challenges, and Future Directions

**DOI:** 10.3390/antibiotics15030305

**Published:** 2026-03-18

**Authors:** Serena Salomè, Chryssoula Tzialla

**Affiliations:** 1 Division of Neonatology, Department of Translational Medical Sciences, University of Naples Federico II, Via Pansini 5, 80131 Naples, Italy; 2 Neonatal Intensive Care Unit, ASST Papa Giovanni XXIII, 24127 Bergamo, Italy; chryssoula.tzialla@asst-pg23.it

**Keywords:** syphilis, congenital syphilis, treatment, mother-to-child transmission

## Abstract

Syphilis remains a global public health concern, with maternal infection posing a substantial risk for congenital syphilis, a preventable condition associated with severe morbidity and mortality. Penicillin, particularly benzathine penicillin G, remains the cornerstone of treatment and the only therapy with proven efficacy in preventing vertical transmission during pregnancy. However, recurrent global shortages, limited manufacturing capacity, mislabeling of penicillin allergy, and the absence of validated alternative regimens for pregnant women and neonates threaten progress toward elimination goals. This review summarizes current evidence on the treatment of syphilis in pregnancy and congenital syphilis, highlighting the established maternal and neonatal regimens, diagnostic and therapeutic challenges, and clinical consequences of delayed or inadequate treatment. We examine the scope and drivers of benzathine penicillin G shortages, the overestimation of penicillin allergy and its impact on care, and the role of neonatal management when maternal therapy is suboptimal. Emerging data on alternative antimicrobial agents, including cephalosporins, tetracyclines, lipoglycopeptides, and novel compounds are discussed considering recent advances in *Treponema pallidum* culture and susceptibility testing. While several non-penicillin agents show promise for non-pregnant populations, robust evidence supporting their use during pregnancy and for the prevention of congenital syphilis is lacking. Addressing these gaps through coordinated supply chain strategies, guideline harmonization, and targeted clinical research is essential to ensure resilient and equitable syphilis control and advance global efforts toward the elimination of congenital syphilis.

## 1. Introduction

Syphilis is a sexually transmitted infection caused by the spirochete *Treponema pallidum* [1]. Vertical transmission from mother to fetus can occur transplacentally at any stage of pregnancy or at delivery, resulting in congenital syphilis (CS), with the highest risk during primary and secondary maternal infection and remaining substantial during latent stages [2]. The probability of transmission is closely related to both the stage of maternal syphilis and the timing of infection acquisition during pregnancy. In untreated women with primary or secondary syphilis in the third trimester, MTFT rates range from 60% to 100%. In contrast, transmission risk is lower in latent stages, estimated at approximately 40% in early latent syphilis and less than 8% in late latent disease. Accurate risk estimation may be complicated in cases where infection predates pregnancy [2].

Untreated or inadequately treated maternal syphilis is associated with adverse pregnancy outcomes that occur in approximately 75% of untreated cases. These outcomes include stillbirth or miscarriage in around 20% of pregnancies and perinatal death in approximately 15%. Additionally, about 20% of infants are born with congenital syphilis, while another 20% present with low birth weight or prematurity requiring admission to a neonatal intensive care unit and multisystem congenital infection [3]. Live-born infants may be asymptomatic at birth or present with early manifestations such as hepatosplenomegaly, anemia, thrombocytopenia, rash, rhinitis, and skeletal abnormalities. Late manifestations include neurologic impairment, sensorineural hearing loss, visual deficits, and characteristic bone and dental deformities [4].

These consequences are preventable with timely testing and diagnosis followed by adequate treatment during pregnancy [5]. Therefore, in 2007, the World Health Organization (WHO) launched the Initiative for the Global Elimination of Congenital Syphilis, defining elimination as a case rate ≤50 per 100,000 live births [6]. Although several countries have achieved validation, global progress has been uneven. Over the past decade, syphilis incidence has resurged globally, affecting both high-income and low- and middle-income countries. According to the last available report by the WHO, syphilis affects over 50 million people worldwide, with 8 million new cases among adults (15- to 49-year-olds) annually. Furthermore, there are 700,000 cases of congenital syphilis, leading to 390,000 syphilis-related adverse birth outcomes and a global congenital syphilis case rate of 523 per 100,000 live births [7].

Universal serological screening for syphilis during pregnancy, a combination of treponemal and non-treponemal tests, is recommended by the latest WHO guidelines to enable early diagnosis and timely treatment, thereby preventing adverse birth outcomes. Most regional and country-specific recommendations are consistent with this guidance [8]. The so-called “reverse” sequence algorithm is frequently recommended because it allows identification of all women with syphilis, including those with latent infections because even non-recent infections must be detected and appropriately treated to prevent congenital syphilis [9].

The effect of untreated syphilis on maternal and neonatal health outcomes is profound, with mother-to-child transmission of syphilis estimated to cost $3.6 million in disability-adjusted life-years and $309 million in medical costs globally [10]. Screening and early detection can reduce these costs because treatment for early-stage syphilis is less expensive than treatment for later stage disease: $41.26 (in year 2001 dollars) compared to $2061.70 for late syphilis [11].

Penicillin remains the only proven therapy capable of treating both maternal infection and fetal disease [9]. However, reliance on a single antimicrobial agent, recurrent global shortages, and the absence of validated alternatives for use in pregnancy threaten elimination efforts. Advances in diagnostics, antimicrobial susceptibility testing, and clinical trial design offer new opportunities to strengthen prevention and treatment strategies.

Risk factors for perinatal syphilis infection operate at multiple levels. At the individual level, they include high-risk sexual behaviors, substance use, belonging to socially or geographically vulnerable populations, poor health literacy, language barriers, stigma, inadequate engagement with healthcare services, and lack of insurance coverage. At the community level, limited access to care, shortages of clinicians, insufficient provider knowledge, stigmatizing attitudes in healthcare settings, and inadequate sexual health education contribute to missed opportunities for prevention and treatment. At the system level, broader structural determinants (such as poverty, systemic racism, homelessness, underfunding of public health infrastructure, insufficient resources for rural and remote areas, and poor integration of health information systems) further exacerbate disparities and increase the risk of congenital syphilis [12]. Therefore, sustained investment in screening, treatment access, and research is essential to achieve and maintain elimination of congenital syphilis.

## 2. Literature Search Strategy

This article was conducted as a narrative review. A structured literature search was performed in PubMed/MEDLINE, Embase, and Scopus to identify relevant studies published up to December 2025. The search strategy combined Medical Subject Heading (MeSH) terms and free-text keywords, including “syphilis”, “congenital syphilis”, “Treponema pallidum”, “pregnancy”, “benzathine penicillin G”, “penicillin allergy”, “antibiotic shortage”, “ceftriaxone”, “doxycycline”, “azithromycin”, “cefixime”, “amoxicillin”, “linezolid”, “dalbavancin”, and “alternative therapy”. Boolean operators (“AND”, “OR”) were used to refine the search.

Additional sources were identified through manual screening of reference lists of selected articles and relevant international guidelines from major public health authorities. Only articles published in English were considered. Priority was given to randomized controlled trials, prospective and retrospective cohort studies, systematic reviews, meta-analyses, and high-quality observational studies. In vitro studies and pharmacokinetic investigations were included when relevant to emerging therapeutic strategies.

Given the narrative design of this review, formal systematic review methodology, predefined inclusion and exclusion criteria, risk-of-bias assessment, and meta-analysis were not performed. Study selection was based on relevance to the topic, methodological quality, and contribution to understanding current therapeutic approaches, challenges in penicillin availability, and potential alternative regimens, particularly in pregnancy and neonatal populations.

## 3. Treatment

### 3.1. Maternal Treatment

Parenteral penicillin has been the cornerstone of syphilis treatment since the 1940s and remains the universally recommended therapy for all stages of infection, including during pregnancy. *T. pallidum* has retained high susceptibility to penicillin, and confirmed resistance has not been documented despite decades of widespread use [13].

Current WHO guidelines recommend intramuscular benzathine penicillin G (BPG) according to the stage of infection: **Primary, secondary, and early latent syphilis**: A single intramuscular dose of 2.4 million units. **Late latent syphilis or syphilis of unknown duration**: Three weekly doses of 2.4 million units (total 7.2 million units). **Neurosyphilis:** Aqueous crystalline penicillin G administered intravenously every 4 h for 10–14 days, as BPG does not achieve treponemicidal concentrations in cerebrospinal fluid [9,14] (as shown in Table 1).

There are other guidelines and recommendations with differences and nuances between them, as shown in Table 2 [9,14,15,16,17,18].

Adequate maternal treatment requires *initiation* of therapy at least four weeks before delivery according to American guidelines [18], *completion* of therapy at least four weeks before delivery according to British guidelines [15], and adherence to recommended dosing intervals. Missed or delayed doses necessitate restarting the full course [9]. Serologic monitoring using non-treponemal tests is employed to assess treatment response. A fourfold decline in titers is considered an adequate response; however, the duration of pregnancy may preclude confirmation before delivery, particularly when treatment is initiated late. A sustained fourfold increase suggests reinfection or treatment failure and requires retreatment [9].

The Jarisch–Herxheimer reaction is due to a release of inflammatory cytokines and occurs in up to 40% of pregnant women, typically manifesting within 1 to 2 h after penicillin administration and resolving spontaneously in 24–48 h. The principal symptoms include chills, myalgia, headache and rash. Symptomatic treatment frequently results in rapid recovery. Although usually self-limited, it may cause transient uterine contractions or fetal distress, warranting short-term fetal monitoring when feasible [19].

### 3.2. Neonatal Treatment

More than 60% of infants with congenital syphilis (CS) are asymptomatic or present with minimal clinical findings at birth, making early diagnosis particularly challenging. As a result, syphilis should be actively considered and excluded in neonates presenting with nonspecific manifestations common to congenital infections, such as fetal growth restriction. As infancy progresses, more characteristic features—including cutaneous lesions, rhinitis, anemia, and thrombocytopenia—may become apparent. Nevertheless, diagnosis based solely on clinical signs is unreliable, underscoring the importance of integrating maternal history, laboratory findings, and the clinical course of both mother and infant into a comprehensive diagnostic assessment [20].

To standardize evaluation and management, the American Academy of Pediatrics has developed a classification system categorizing congenital syphilis as “proven or highly probable,” “possible,” “less likely,” or “unlikely,” based on clinical findings and maternal treatment history. As previously reported in detail, maternal treatment is defined inadequate when: it is initiated/completed less than 4 weeks before delivery, it is based on non-penicillin regimens and/or a lack of a 4-fold decrease in maternal non-treponemal titers is observed. Clinically, CS is further categorized into early congenital syphilis, with manifestations occurring before two years of age, and late congenital syphilis, with symptoms emerging thereafter [17,18]. This risk stratification underpins current CDC (Centers for Disease Control and Prevention) and WHO recommendations and informs decisions regarding diagnostic evaluation and therapeutic intensity, as shown in Table 3, such as differences with other available recommendations.

Aqueous crystalline penicillin G may be associated with complications such as phlebitis, and each vial contains a measurable potassium load, which may be clinically relevant in vulnerable neonates. If any doses are missed during the prescribed course, a full 10-day regimen must be restarted. Ampicillin is not considered an adequate substitute for penicillin G in the treatment of congenital syphilis. In neonates treated with ampicillin for presumed sepsis, an additional full course of aqueous crystalline penicillin G is required once CS is diagnosed or strongly suspected [18].

The Jarisch–Herxheimer reaction may occur even in neonates within 24 h of treatment initiation and is characterized by tachycardia or tachypnea, in addition to symptoms still described for the mothers. Management is supportive, and symptoms typically resolve rapidly. In cases of penicillin allergy, desensitization is recommended. During a period of penicillin shortages in Brazil in 2015, the alternative treatment regimen recommended for infants included ceftriaxone 25 to 50 mg/kg, once a day, intravenously or intramuscularly for 10 to 14 days. Another treatment was based on cefazolin for 10 days. These alternative treatments for infants were proposed without scientific evidence demonstrating their effectiveness [21]. Moreover, ceftriaxone is contraindicated in premature infants and neonates with hyperbilirubinemia and should not be administered concomitantly with intravenous calcium-containing solutions due to the risk of fatal precipitates.

Late congenital syphilis, diagnosed after infancy, requires weight-based intravenous aqueous crystalline penicillin G administered every 4–6 h for 10 days. Management of late CS is associated with additional challenges, including prolonged hospitalization and the potential for irreversible sequelae. These burdens reinforce the critical importance of prevention strategies aimed at early identification and treatment of maternal infection [18].

Careful follow-up is a critical component of management. Infants with reactive nontreponemal test results at birth should undergo serological monitoring every 2–3 months to confirm progressive decline and eventual normalization of antibody titers. Passively acquired maternal nontreponemal antibodies may persist for up to 15 months; however, in most uninfected infants, titers typically become nonreactive by 6 months of age [22]. Persistence of elevated titers beyond 6 months warrants further clinical evaluation and consideration of treatment [18]. Failure to ensure appropriate follow-up remains a significant challenge. In some regions of the United States, loss to follow-up rates have reached up to 65%, increasing the likelihood of missed or delayed diagnoses and subsequent neonatal complications [23]. In contrast, lower rates have been reported in other healthcare settings, potentially reflecting differences in healthcare system organization; for example, a recent Italian study documented a 12.5% loss to follow-up rate [24]. Prolonged and structured monitoring is essential, as children with congenital syphilis remain at risk for late-onset and persistent sequelae, including intellectual disability, hearing impairment, and skeletal abnormalities [20].

## 4. Challenges

The management of syphilis in pregnancy and congenital syphilis faces several interrelated challenges. Chief among these is the near-total reliance on benzathine penicillin G without validated alternatives for use during pregnancy. Recurrent global shortages of BPG have been reported across diverse settings and have resulted in missed or inadequate treatment of pregnant women.

### 4.1. Guidelines Heterogeneity

As shown above and summarized in the specific tables, available guidelines present several differences that could lead to more difficult management of maternal and neonatal infection. For instance, the definition of inadequate maternal treatment based on *initiation* [18] or *completion* [15] of therapy at least four weeks before delivery. A single word leads to completely different definition of neonatal risk and subsequent management of laboratory evaluation and treatment.

Furthermore, heterogeneity has been highlighted in the management of missing doses, alternative drugs for shortages and/or allergy. Therefore, there is an urgent need for harmonization because of possible consequences and implications.

### 4.2. Multidisciplinary Working

Management of syphilis infection during pregnancy requires input from multiple specialties; therefore, effective multidisciplinary collaboration is essential. Clear and timely communication, together with strong interprofessional relationships, is fundamental to delivering high-quality care. The composition of the multidisciplinary team varies according to local organization and case load but may include screening team and/or specialist Midwife, Genitourinary Medicine services, Pediatrics and Neonatology, Obstetrics, Microbiology and/or Virology. Every instance of syphilis in pregnancy should be managed collaboratively. Members of the screening team play a pivotal role in coordinating care and ensuring the effective functioning of the multidisciplinary team. Close collaboration with the screening laboratory is equally important. Clear communication pathways must be in place to guarantee that positive results are promptly conveyed to screening midwives in accordance with national screening standards. Strong laboratory links are fundamental to ensuring appropriate clinical management and timely interventions in partnership with the patient [15].

Care planning is another critical component. As suggested by British recommendations [15], a Syphilis Birth Plan should be implemented in all confirmed cases of syphilis during pregnancy facilitating the transfer of essential information between professionals regarding maternal treatment and neonatal management at birth. A neonatal alert system, or a comparable mechanism, should be established to ensure that relevant healthcare professionals are directed to the care plan at the time of delivery.

### 4.3. Shortage

The absence of proven alternatives to penicillin for the treatment of syphilis during pregnancy makes uninterrupted access to BPG critical, particularly in high-incidence settings. Ensuring adequate national and regional control of penicillin supplies is essential to prevent cases of congenital syphilis resulting from delayed, interrupted, or inadequate maternal treatment [25,26]. Nevertheless, BPG availability has been compromised by periodic and prolonged shortages reported across high-income, middle-income, and low-income countries [27]. The inability to treat pregnant women during these shortages has been estimated to contribute to more than half a million cases of congenital syphilis worldwide [26,28].

Global supply constraints are driven largely by structural market failures. A competitive market characterized by low profit margins and limited visibility of global demand has led to a progressive reduction in the number of manufacturers producing the active pharmaceutical ingredient (API). By 2016, only three API manufacturers, all based in China, remained active in supplying the global market. Persistent imbalances between supply and demand have resulted in recurrent shortages, underscoring substantial market vulnerability and the urgent need for coordinated mitigation strategies [29]. At the national level, strengthening demand forecasting, procurement planning, stock monitoring, and regulatory oversight of authorized suppliers is critical. At the global level, enhanced market transparency, early-warning systems for shortages, and continuous monitoring of supply chain risks are required. Additionally, improving incentives to produce quality-assured BPG and ensuring affordability remain central to long-term supply stability [29].

Between 2014 and 2016, at least 39 countries reported shortages of BPG [27], leading to the use of non-recommended treatment regimens and a subsequent increase in congenital syphilis cases [21,26]. Similar patterns have been observed more recently, with ongoing global shortages affecting more than 39 countries worldwide, including both high-income nations and low- and middle-income countries [25,27]. In several settings, pregnant women with syphilis were either left untreated or received alternative medications for which efficacy data in preventing congenital syphilis are lacking, including during documented shortages in Brazil and other countries [27].

In the United States, persistent BPG shortages have been driven by supply chain fragility and rising demand. Injectable BPG is supplied by a single manufacturer, rendering the national supply highly vulnerable to disruptions. Product recalls involving tens of thousands of adult syringes have necessitated production shifts, resulting in substantial backorders of pediatric formulations projected to extend into late 2025, alongside ongoing constraints affecting adult doses. Because penicillin G is the only evidence-based therapy for syphilis during pregnancy, delays in access have translated directly into preventable cases of congenital syphilis. Moreover, limited transparency regarding product availability and distribution has often favored large hospital systems over safety-net clinics, exacerbating inequities in access [30].

Several policy measures have been proposed to address these challenges. Inclusion of penicillin G on the Strategic Active Pharmaceutical Ingredients Reserve list could enhance national preparedness, while use of the Defense Production Act may support increased manufacturing through loans, purchase guarantees, and other financial incentives [30]. Additional strategies include enforcing fair-allocation frameworks to protect clinics serving pregnant women during shortages, mandating manufacturer reporting on production and distribution, and aligning supply with public health needs through improved coordination among healthcare systems and regulatory agencies. Investment by the National Institutes of Health and the Biomedical Advanced Research and Development Authority in clinical trials evaluating alternative therapies safe for use during pregnancy has also been recommended [30].

Expanded antenatal screening, strengthened partner services, secure access to treatment, and financing reforms are essential complementary strategies that could help re-establish congenital syphilis as a rare diagnosis [30]. However, shortages have also been associated with medication errors, including inappropriate intramuscular administration of aqueous crystalline penicillin, a short-acting formulation unsuitable for this indication [31].

Finally, future demand for BPG is expected to increase substantially. Expanded use of point-of-care testing is projected to raise the number of doses required for pregnant women from approximately 414,459 in 2019 to more than 1,078,428 by 2030 [32]. This anticipated growth further emphasizes the urgency of strengthening global supply chains and prioritizing BPG availability as a cornerstone of congenital syphilis elimination efforts.

### 4.4. Allergy

Penicillin allergy is the most frequently reported drug allergy and is reported by approximately 6% to 25% of pregnant individuals depending on the setting [33]. Importantly, true IgE-mediated reactions to penicillin are rare and true penicillin allergy is estimated to affect fewer than 3% of individuals overall [33]. Penicillin-related anaphylaxis occurs in approximately 1 per 100,000 administrations (range 0–3), while any adverse reactions are reported in roughly 2 per 1000 patients [34]. It has been estimated that up to 90% of self-reported penicillin allergies are incorrectly labeled, frequently due to confusion between drug intolerance, coincidental symptoms, or manifestations of infectious disease rather than true allergic responses [35]. These data indicate that the perceived risk of penicillin allergy substantially exceeds the actual incidence of severe reactions. Addressing this widespread misclassification is particularly important in pregnancy, where therapeutic alternatives are limited as oral doxycycline that is contraindicated because of concerns regarding potential teratogenic effects [14].

Mislabeling of penicillin allergy has been associated with significant clinical and public health consequences, including suboptimal antibiotic selection, increased use of broad-spectrum agents, higher rates of antimicrobial resistance, increased incidence of *Clostridioides difficile* colonization and infection, prolonged hospital stays, and higher healthcare costs. Evidence suggests that most individuals who carry a penicillin allergy label can safely receive penicillin or related β-lactam antibiotics following appropriate assessment [33].

Accurate assessment of suspected penicillin allergy relies on a detailed clinical history, which is essential for distinguishing true hypersensitivity reactions from non-allergic adverse events or symptoms attributable to the underlying infection. Careful clinical evaluation is critical, as many reported reactions lack features predictive of immune-mediated allergy.

**Figure 1:**
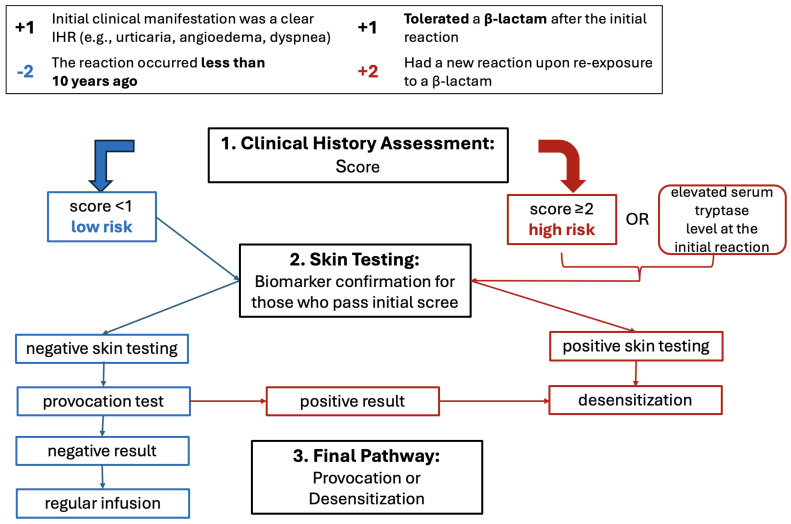
Immediate Hypersensitivity Reaction Management [36].

In this context, penicillin desensitization represents a key management strategy for pregnant women with syphilis who have a history suggestive of immediate hypersensitivity and is recommended by the main guidelines, as shown in Table 1 and Table 2. Recommended approaches involve risk stratification based on clinical history and, when available, skin testing. Patients categorized as high risk are directed toward rapid desensitization protocols, which typically involve gradual intravenous dose escalation over approximately four hours to achieve a cumulative dose of around 1,000,000 IU of potassium penicillin G. Patients assessed as low risk may undergo drug provocation testing using a stepwise graded challenge, often beginning with intravenous crystalline penicillin G and, in some protocols, transitioning to oral amoxicillin to improve tolerability and feasibility [36]. These strategies allow safe re-exposure to penicillin in most cases and are summarized in Figure 1.

Patients with a history of severe delayed hypersensitivity reactions, such as Stevens–Johnson syndrome, should not undergo desensitization and instead require specialist evaluation by an allergist [17].

### 4.5. Alternative Treatment

Only a limited number of antibiotics have been evaluated in randomized controlled trials (RCTs) for the treatment of early syphilis [37]. The identification and evaluation of alternative treatments for syphilis have been explicitly recognized by the World Health Organization as a research priority [9]. The public health implications of expanding the therapeutic armamentarium are substantial, as effective alternatives could reduce the global burden of disease and support international goals for the elimination of mother-to-child transmission of syphilis [38]. Validated non-penicillin regimens would ensure continuity of care during periods of benzathine penicillin G (BPG) shortages, in individuals with confirmed penicillin allergy, or in settings where access to injectable penicillin is limited or impractical.

When alternative regimens are used during pregnancy—whether because of penicillin allergy or drug shortages—careful postnatal evaluation of the newborn is essential, and treatment for congenital syphilis should be considered as indicated [17]. International guidance on this issue remains heterogeneous. A review of national and regional guidelines revealed substantial discrepancies in recommended therapeutic approaches, with alternative regimens to BPG included in 42 guidelines (68%), predominantly from African and Asian countries; notably, only 20 of these guidelines explicitly acknowledged that non-penicillin regimens have not been proven effective in treating fetal infection [8]. This inconsistency may contribute to inappropriate maternal management and inadequate neonatal prevention strategies.

## 5. Future Perspectives

Several non-penicillin antibiotic strategies have demonstrated efficacy comparable to benzathine penicillin G (BPG) for the treatment of non-neurological syphilis in non-pregnant adults, including individuals living with HIV. Ceftriaxone, azithromycin, and doxycycline monotherapies have shown similar serologic cure rates to BPG in early syphilis and may reduce reliance on penicillin in settings where its use is not feasible [13]. Nevertheless, penicillin remains indispensable for pregnant women and for the prevention of congenital syphilis, as it is the only therapy proven to reliably cross the placenta and eradicate fetal infection [14].

**Ceftriaxone**, a third-generation cephalosporin, is an effective alternative for early syphilis and neurosyphilis in non-pregnant adults and current CDC guidelines recommend ceftriaxone 1 g daily either IM or IV for 10 days as an alternative for primary and secondary syphilis or ceftriaxone 1 to 2 g daily either IM or IV for 10 to 14 days as an alternative for neurosyphilis in nonpregnant adults; it is not mentioned as an option for latent syphilis [17]. However, its requirement for daily intramuscular or intravenous administration for 10–14 days limits feasibility and compliance. Moreover, even though it is generally considered safe in pregnancy [17], there are limited data about its use in this population. In fact, it was reported in 14 individual case reports involving 166 pregnant patients with syphilis [39] with a recent retrospective analysis involving a group of 79 pregnant women [40] with safety profile, even though robust pharmacologic and clinical data are lacking. Moreover, none of the infants described were diagnosed with congenital syphilis after its use, but regimens differed, and long-term follow-up was often lacking and the data about children were available only for 13 cases [39]. Therefore, ceftriaxone may be considered as an option in pregnancy if IM and IV penicillin are unavailable. The optimal dose and duration of ceftriaxone use during pregnancy are not defined. For pregnant individuals with primary or secondary syphilis, the recommended regimen for nonpregnant persons (1 g daily either IM or IV for 10 days for early syphilis) might be considered, but both patient and provider should be aware of the uncertainties and limitations of the data. The optimal duration of ceftriaxone therapy for late latent syphilis is not clear but, based on the duration recommendations for penicillin, it would likely be 21 to 28 days [39].

**Doxycycline** is recommended for non-pregnant adults but has historically been avoided in pregnancy because of concerns regarding teratogenicity and dental staining. Although these concerns are increasingly questioned, the absence of efficacy data for preventing congenital syphilis currently precludes its use during pregnancy [41,42]. **Minocycline** may represent another tetracycline option for early syphilis, but evidence is sparse and absent in pregnant populations [43]. There are only a few case reports and one small case series evaluating the use of any tetracycline in pregnancy involving 14 patients as a whole and only one of their infants was diagnosed with congenital syphilis at 10 weeks of life but tetracycline was given late during pregnancy, and it was unknown when the pregnant person contracted syphilis so conclusions are limited [39].

Oral **amoxicillin**, alone or combined with probenecid, has shown high efficacy in randomized trials among patients with HIV infection [44]. However, outcomes in pregnancy have been inconsistent [45,46,47], with acceptable results in early syphilis with 33 cases treated with amoxicillin or ampicillin with or without probenecid resulted in no infants born with congenital syphilis but unacceptably high rates of congenital syphilis when used for late disease with 52 late syphilis cases resulted in 15 infants with congenital syphilis, all from pregnant women treated with amoxicillin (1500 mg/d) as reported in a single study [46]. Consequently, amoxicillin is not currently recommended during pregnancy [39], although high-dose regimens are under investigation in a clinical trial enrolling 20 adult pregnant women in good health for pharmacokinetic study (ClinicalTrials.gov number, NCT05309928 https://clinicaltrials.gov/study/NCT05309928 (accessed on 7 March 2026)).

**Azithromycin** (2 g in single oral dose), once considered an alternative for early syphilis [37], is no longer recommended due to widespread macrolide resistance and high treatment failure rates, including documented cases of congenital syphilis and neonatal deaths [48,49].

**Cefixime**, an oral third-generation cephalosporin that is widely available, low cost, and considered safe in pregnancy, has emerged as one of the most promising candidates. Early trials demonstrated treatment responses comparable to penicillin, and ongoing randomized controlled trials are evaluating a 10-day cefixime regimen for early syphilis [50,51]. Interim analyses suggest similar efficacy between cefixime and penicillin, supporting continued evaluation [52,53]. Data about its use in pregnant women with syphilis are not currently available.

**Linezolid** has demonstrated in vitro activity against *Treponema pallidum* [54], but clinical studies have shown limited efficacy with short regimens, and concerns regarding resistance have tempered enthusiasm [55]. Ongoing studies are investigating whether longer regimens could address this limitation [56]. Human pregnancy data do not indicate teratogenicity, but experience remains limited [57]. Data about its use in pregnant women with syphilis are not currently available.

**Dalbavancin** represents a particularly innovative candidate due to its long half-life and potential for single-dose therapy. In vitro studies demonstrate potent anti-treponemal activity, with plasma concentrations exceeding minimum inhibitory concentrations for several weeks, suggesting sustained therapeutic exposure after a single infusion [58]. This pharmacologic profile makes dalbavancin attractive for further investigation, particularly in populations where adherence to multi-dose regimens is challenging. Data about its use in pregnant women with syphilis are not currently available.

**Zoliflodacin**, developed for *Neisseria gonorrhoeae*, has also shown in vitro activity against *Treponema pallidum* [59], likely due to conserved targets within the DNA gyrase B subunit [60]. Data about its use in pregnant women with syphilis are not currently available.

Despite these advances, most promising alternatives remain investigational, parenteral, or insufficiently studied in pregnancy. Randomized controlled trials evaluating efficacy, safety, and optimal duration of alternative regimens in pregnant women and neonates are urgently needed. Expanding the therapeutic armamentarium is essential to ensure resilience of syphilis treatment strategies in the face of recurrent penicillin shortages and evolving public health demands.

**Figure 2:**
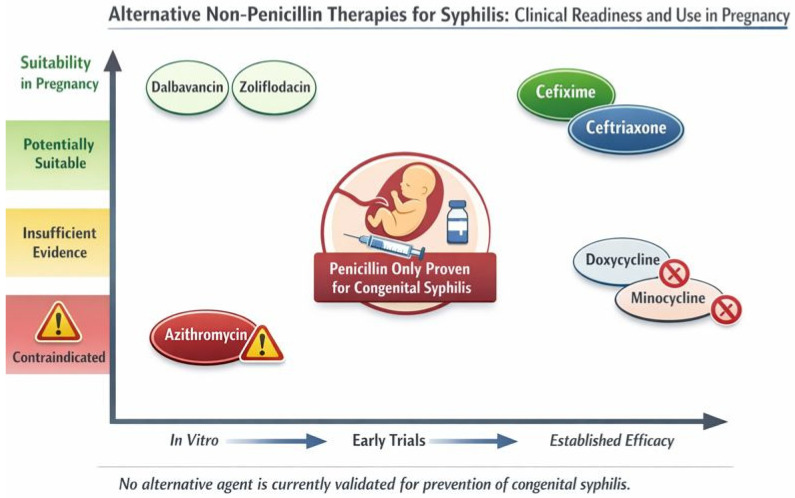
Alternative therapies in pregnancy.

The use of these alternative drugs is represented in Figure 2.

## 6. Conclusions

Congenital syphilis is a preventable yet persistently resurgent condition that reflects broader vulnerabilities in health systems, drug supply chains, and research prioritization. Despite decades of effective therapy, syphilis management remains uniquely dependent on penicillin, with benzathine penicillin G serving as the sole evidence-based treatment capable of curing maternal infection and reliably preventing fetal disease. Recurrent global shortages, limited manufacturing capacity, inequitable distribution, and misclassification of penicillin allergy have exposed the fragility of this single-drug paradigm and have directly contributed to preventable adverse pregnancy and neonatal outcomes.

This review underscores that, while several alternative antibiotics demonstrate efficacy for early syphilis in non-pregnant adults, none currently match penicillin’s proven effectiveness in pregnancy or for congenital syphilis prevention. Discrepancies among international guidelines and the use of non-validated regimens further complicate clinical decision-making and may inadvertently increase neonatal risk. Advances in *Treponema pallidum* culture and antimicrobial susceptibility testing represent a major scientific turning point, opening the door to the systematic evaluation of novel and repurposed agents, including long acting and oral therapies that could transform syphilis care if proven safe and effective in pregnancy.

Moving forward, the elimination of congenital syphilis will require a multipronged strategy: securing stable and transparent supplies of benzathine penicillin G; improving penicillin allergy assessment and access to desensitization; optimizing existing treatment regimens to improve adherence and conserve resources; and investing in randomized controlled trials that include pregnant women and neonates. Without expanding the therapeutic armamentarium and strengthening implementation frameworks, progress toward elimination targets set by the World Health Organization will remain vulnerable. Ensuring resilient, evidence-based treatment strategies is therefore not only a clinical imperative but a global public health priority.

## Figures and Tables

**Table 1:** Summary of the updated recommendations on treatment of *Treponema pallidum* (syphilis) during pregnancy [9].

Recommendations	Strength of Recommendation and Certainty of Evidence
**Early syphilis (primary, secondary and early latent syphilis of not more** **than two years’ duration) in pregnant women**	
In pregnant women with early syphilis, the **WHO recommends**: BPG 2.4 million units once intramuscularly.	**Strong recommendation**, very low certainty in evidence of effects *(updated 2023)*
If benzathine penicillin is not available, the **WHO suggests**: procaine penicillin 1.2 million units intramuscularly once daily for 10 days. In **rare situations** when benzathine or procaine penicillin cannot be used (e.g., due to confirmed penicillin allergy, which occurs in less than 3% of the population, and where penicillin desensitization is not possible) or are not available (e.g., due to stock-outs), the **WHO suggests** one of the following options with caution and enhanced follow-up: ceftriaxone 1 g intramuscularly once daily for 10–14 days; erythromycin 500 mg orally four times daily for 14 days. *Remarks*: If the stage of syphilis is unknown, follow recommendations for pregnant women with late syphilis. Although erythromycin treats the pregnant woman, it does not cross the placental barrier completely and as a result the fetus is not treated. Therefore, it is necessary to treat the newborn infant soon after delivery [14]. Doxycycline is contraindicated during pregnancy.	**Conditional** **recommendation**, very low certainty in evidence of effects *(updated 2023)*
**Late syphilis (late latent and tertiary syphilis of more than two years’** **duration without evidence of treponemal infection) or unknown duration in** **pregnant women**	
In pregnant women with late syphilis or an unknown duration of infection, the **WHO recommends**: BPG 2.4 million units intramuscularly once weekly for three consecutive weeks. *Remark*: The interval between consecutive doses of BPG should not exceed 14 days.	**Strong recommendation**, very low certainty in evidence of effects *(updated 2023)*
If benzathine penicillin is not available, the **WHO suggests**: procaine penicillin 1.2 million units intramuscularly once daily for 20 days. In **rare situations** when benzathine or procaine penicillin cannot be used (e.g., due to confirmed penicillin allergy, which occurs in less than 3% of the population, and where penicillin desensitization is not possible) or are not available (e.g., due to stock-outs), the **WHO suggests** using, with caution and enhanced follow-up: erythromycin 500 mg orally four times daily for 30 days. *Remarks*: Although erythromycin treats the pregnant woman, it does not cross the placental barrier completely and as a result the fetus is not treated. Therefore, it is necessary to treat the newborn infant soon after delivery [14]. Doxycycline is contraindicated during pregnancy.	**Conditional** **recommendation**, very low certainty in evidence of effects *(updated 2023)*

**Table 2:** Maternal Syphilis Treatment Recommendations.

Stage/Condition	WHO (2024 Update) [9]	European Guideline (2020) [16]	BASHH UK (2024) [15]	CDC (2021) [18]
**Early Syphilis** (Primary, Secondary, Early Latent)	**BPG** 2.4 million units (MU) IM in a single dose.	**BPG** 2.4 MU IM in a single dose.	**BPG** 2.4 MU IM; considers a **2nd dose** 1 week later if in the 3rd trimester.	**BPG** 2.4 MU IM once; some evidence supports a **2nd dose** 1 week later to prevent CS.
**Late Syphilis** (Late Latent, Unknown, Tertiary)	**BPG** 2.4 MU IM weekly for **3 consecutive weeks**.	**BPG** 2.4 MU IM weekly on Days 1, 8, and 15.	Follows BASHH national guidelines; assumes late syphilis if history is unclear.	**BPG** 7.2 MU total (3 doses of 2.4 MU at 1-week intervals).
**Alternative if BPG Unavailable**	**Procaine penicillin** 1.2 MU IM daily (10 days for early; 20 days for late).	**Procaine penicillin** 600,000 units IM daily (10–14 days for early; 17–21 days for late).	**Ceftriaxone** (limited data).	No proven alternatives: procaine penicillin is an option for non-pregnant adults, but BPG is preferred.
**Penicillin Allergy**	**Desensitization** preferred. Alternatives (with caution): **Ceftriaxone** or **Erythromycin**.	**Desensitization** followed by standard penicillin regimen.	**Desensitization** should be considered. Urgent allergy testing is required.	**Desensitization** and treatment with penicillin is the **only** documented effective therapy.
**Special Notes**	**Azithromycin** is specifically **deleted** as a recommended alternative.	Missing a dose by **>14 days** in late syphilis requires restarting.	Routine use of steroids to prevent Jarisch–Herxheimer reaction is **not recommended**.	Missed doses **>9 days** are unacceptable for late syphilis; the full course must be repeated.

**Table 3:** Neonatal/Congenital Syphilis Recommendations for Treatment and Management.

Scenario of CS [17,18]	Key Characteristics CDC (2021)/AAP Red Book (2024) [17,18]	Recommended Actions		
		CDC (2021)/AAP Red Book (2024) [17,18]	European Guideline (2020) [16]	BASHH UK (2024) [15]
Confirmed proven or highly probable	Abnormal physical exam OR Neonatal RPR titer ≥4x mother’s	**Aqueous crystalline penicillin G** 100,000–150,000 units/kg/day IV (administered every 12 h then every 8 h) for **10 days** or **Procaine Penicillin** G IM	**Aqueous crystalline penicillin G** 150,000 units/kg IV daily (6 doses every 4 h) for **10–14 days**. If CSF is normal: **BPG** 50,000 units/kg IM (single dose) or **procaine penicillin** daily for 10–14 days.	Infants at “high risk”: **Aqueous crystalline penicillin G** 25 mg/kg (≈41,700 UI/kg) IV for **10 days**. Frequency: every 12 h if <7 days old; every 8 h if 7–28 days old, every 6 h if >28 days old.
Possible	Normal physical exam AND Neonatal RPR titer <4x mother’s, BUT mother was inadequately treated	**Aqueous crystalline penicillin G** (IV) or **Procaine penicillin** (IM) for 10 days. Single-dose **BPG** only if full evaluation is normal and follow-up certain.	*Not described*	
Less likely	Normal physical exam, Neonatal RPR titer <4x mother’s AND Mother adequately treated during pregnancy	No treatment and close follow-up OR single dose of **BPG** 50,000 units/kg IM once.	*Not described*	Infants at “low risk” require serology and no treatment
Unlikely	Normal physical exam AND RPR titer <4x mother’s AND Mother adequately treated before pregnancy with stable low titers	No treatment required. If follow-up is uncertain, consider single dose of **BPG** IM	*Not described*	No need for the neonate to undergo testing for syphilis.
**Penicillin Allergy Alternatives**		**Ceftriaxone** can be considered with caution in jaundiced neonates if penicillin is unavailable, but data are insufficient.	No specific alternative listed; desensitization is implied for required treatment.	**Ceftriaxone** (75–100 mg/kg IV once daily for 10–14 days) if hospital admission is impossible.
**Follow-up Protocol**		RPR/VDRL every 2–3 months until nonreactive.	*Not described*	RPR at 3 months: if negative, discharge; if positive but falling, repeat at 6 months; unchanged from birth or rising or IgM positive, refer to pediatric infection specialists

Different colors, from red to green, highlight the different risks as in a traffic light.

## Data Availability

No new data were created or analyzed in this study. Data sharing is not applicable to this article.

## Abbreviations

The following abbreviations are used in this manuscript:

CS	congenital syphilis
WHO	World Health Organization
BPG	benzathine penicillin G
CDC	Centers for Disease Control and Prevention
AAP	American Academy of Pediatrics
RPR	rapid plasma reagin
IV	intravenous
IM	intramuscular
API	active pharmaceutical ingredient
RCT	randomized controlled trial
